# Effectiveness of Chlorhexidine and Aloe Vera Mouthwash in Patients With Periodontal Disease: A Randomized Controlled Trial

**DOI:** 10.7759/cureus.66675

**Published:** 2024-08-12

**Authors:** Nasreen Hamarash Hamonari

**Affiliations:** 1 Dental Public Health, Kurdistan Higher Council of Medical Specialists, Erbil, IRQ

**Keywords:** gingivitis placebo, dental plaque, randomized clinical trial, gingival index, mouthwashes, plaque index, periodontal disease, chlorhexidine, aloe vera

## Abstract

Background: Aloe vera has gained significant attention in clinical research, and promoting natural substances is a prevailing trend in dentistry.

Aim: This study compares the effectiveness of aloe vera mouthwash and 0.2% chlorhexidine gluconate mouthwash in reducing plaque accumulation and gingivitis.

Materials and methods: A single-masked trial included 270 volunteers who were systemically healthy and aged between 18 and 45 years. The participants were randomly assigned to three groups: Group A (the test group) received aloe vera mouth rinse, Group B (the positive control group) received a placebo (distilled water), and Group C (the negative control group) received 0.2% chlorhexidine. Clinical indicators, which include the plaque index (PI) by Sillness and Loe in 1964 and the gingival index (GI) by Loe and Sillness in 1963, were evaluated at baseline, day 15, and day 30 for all three groups. Participants were directed to rinse their mouths with the specified mouthwash twice daily for 30 days.

Results: Significant reductions in the GI and PI were observed in both aloe vera and chlorhexidine mouthwashes, with a statistical significance of p<0.001. The placebo mouthwash also showed reductions in both the GI and PI, with a significance level of p<0.001. Post hoc analysis revealed no significant differences between the aloe vera and chlorhexidine groups for the GI and PI, with p-values of 0.6 and 0.8, respectively.

Conclusion: Aloe vera has proven equally effective as chlorhexidine in reducing plaque and gingivitis. This makes it a viable alternative for treating and preventing gingivitis, appealing to those preferring natural, holistic oral care. Incorporating aloe vera mouthwash into daily routines offers an effective, natural solution for maintaining gum health.

## Introduction

Herbal and natural products are increasingly used in dentistry to treat oral diseases and conditions. Numerous plant-based solutions have antibacterial properties and are highly effective at teeth cleaning. For comparable reasons, these are valuable alternatives to traditional antibiotics and antimicrobials [1-4].

Maintaining periodontal health is a crucial part of total dental health. Plaque-induced gingivitis, which affects more than 90% of the population, highlights the importance of preventive oral care [5]; if not properly treated, plaque-induced gingivitis can develop into periodontal disease. Symptoms of gingivitis caused by plaque include swollen and red gums and bleeding easily during brushing or flossing. When inflammation spreads to the supporting structures of teeth, it can cause bone defects in the periodontal area, ultimately leading to tooth loss [6]. The primary objective in preventing periodontal diseases is to control plaque formation and manage gingivitis through individual oral hygiene practices and regular professional evaluations and maintenance when necessary [7]. However, completely eradicating bacterial plaque presents challenges, making it essential to reduce plaque accumulation or modify its composition to prevent periodontal disease [8].

It is necessary to properly control plaque using mechanical and chemical approaches to prevent periodontal diseases. Chlorhexidine is considered the most effective method for chemical plaque treatment because of its strong antibacterial properties and ability to decrease plaque buildup. Long-term use of chlorhexidine is limited because of its adverse effects, such as discoloration of dental materials and teeth, erosion of the tongue and mucosa, a peculiar or unpleasant taste in the mouth, and increased buildup of supragingival calculus [9,10].

As concerns grow over the harmful effects of chemical mouthwashes, there is a growing interest in exploring herbal alternatives with antibacterial and anti-inflammatory properties. Aloe vera mouthwash is one such option that can aid in controlling plaque and treating gingivitis, with the added advantage of having no discernible side effects [2,10].

The pharmacological advantages of aloe vera are derived from its multifaceted characteristics, including wound-healing, immunomodulatory, anti-inflammatory, antioxidant, and antimicrobial features [11]. Furthermore, aloe vera exhibits properties that reduce edema by inhibiting matrix metalloproteinases, blocking obstruction of polymorphonuclear leukocyte (PMN) release, and modulating the cyclooxygenase and lipoxygenase pathways. Consequently, activated PMNs inhibit free oxygen radical activity [12].

Although the therapeutic attributes of aloe vera have been widely recognized, there is a need for additional scholarly research on its utilization as a dental mouthwash. This study compares aloe vera and chlorhexidine mouthwash to analyze their impact on periodontal health. It aims to provide insights into their benefits in promoting oral hygiene and preventing periodontal diseases.

## Materials and methods

The study was a 30-day, single-masked, randomized, parallel, controlled clinical trial designed to compare the effectiveness of aloe vera mouthwash (KIN, Laboratorios KIN, Barcelona, Spain, 500 milliliters) and 0.2% chlorhexidine gluconate mouthwash (500 milliliters) in reducing plaque and relieving gingival inflammation. Participants were randomly assigned to three groups using a lottery method and were identified by code numbers. The study included 270 systemically healthy individuals with moderate to severe gingivitis, comprising 144 females and 126 males aged 18 to 45. They were randomly selected from the outpatient departments of the Azadi and Hawler Centers in Erbil City, Kurdistan Region, Iraq. The study was conducted from April 1 to October 1, 2023.

The Ethical and Scientific Committee of the Kurdistan Higher Council of Medical Specialties approved the study in a letter dated August 12, 2022, with reference number 2276. The volunteers were fully informed about the methodology employed in the clinical trial before providing their informed written consent. The participants also received sufficient information about their ability to withdraw from the study at any time during its conduct.

Participants in good health who had not previously received treatment for plaque-induced gingivitis were evaluated for potential inclusion in the research study. Eligibility criteria included demonstrating signs of moderate to severe gingivitis with a gingival index (GI) score >1, being between 18 and 45 years old, having at least 20 natural teeth (with a minimum of five in each quadrant), and agreeing to participate by signing a consent form. Exclusion criteria encompassed factors such as probing pocket depths of 3 mm or more, recent antibiotic or anti-inflammatory dental product use, smoking, pregnancy, or medical conditions that could impact the study. Additionally, individuals with recent periodontal treatment or known allergies to study substances were also not eligible for participation.

The data-gathering procedure used a pro forma split into two sections. The initial element of the proforma included a structured interview segment that collected data on demographic information, oral hygiene habits, and the participants' medical and dental backgrounds. This section aimed to gather relevant background information on the participants. Part two of the proforma comprised the clinical assessment of periodontal health. The study reported the following clinical parameters: The plaque index (PI), developed by Silness and Loe in 1964 [13], quantifies the degree of plaque buildup on teeth, and the GI by Loe and Silness in 1963 [14], assessed the extent of gingivitis by investigating the state of gingival tissue variables, including redness, edema, and bleeding.

All participants were baseline assessed before the mouthwashes were administered. The efficiency of the mouthwashes was evaluated by examining follow-up after 15 and 30 days, considering the dependent variables. The tools utilized to record indices included a mouth mirror, an explorer, a periodontal probe, and tweezers. Four surfaces (buccal, lingual/palatal, mesial, and distal) of six specified index teeth (18, 23, 26, 38, 43, 46) were assessed throughout the examination.

An evaluation was performed on the dental PI and GI by inspecting the four surfaces of the index teeth to determine the presence or absence of signs linked to these indices. A calibrated periodontal probe was utilized to evaluate the sites during the examination, with a 10-second interval allowed to confirm the presence or absence of gingival bleeding. Plaque detection was done by directly observing or detecting the soft matter deposited along the gingival margin and in the tooth and gingival pocket. Plaque index scores of two and three were assigned accordingly. Dental plaque was deemed present if the distinctive indication was observed in at least one location. The PI was classified as follows: healthy = PI <1; fair = PI 1-1.9; bad = PI ≥2.

Gingivitis was diagnosed if there was bleeding at a single site during the inspection, corresponding to scores two and three on the GI index. The GI quantified the severity of gingivitis on a scale ranging from 0.1 to 3.0, with specific classifications: 0.1-1.0, mild gingivitis; 1.1-2.0, moderate gingivitis; and 2.1-3.0, severe gingivitis. Gingivitis was classified as moderate or severe if the GI exceeded one, computed as the average value of all tooth surfaces assessed.

The size of the sample was determined using a power of 0.80, an effect size 𝑓≈0.23, and α=0.05 (95% confidence level), k = 33 (number of groups), according to the formula: N = f2(k1) (Zα/2​+Zβ​)2. The sample size calculation resulted in an estimated N≈296.33, rounded to 324, to account for an anticipated 10% dropout rate.

From the 324 participants initially screened, 300 individuals with gingivitis were selected and enrolled in the clinical study, adhering to the specified inclusion and exclusion criteria. One week before the commencement of the study, participants received professional dental cleaning (scaling and polishing) and were instructed to maintain their usual oral care routine. However, by the end of the first week, 30 participants withdrew from the study for various reasons.

A total of 270 volunteers, comprising 126 males and 144 females, were randomly allocated to three groups using a computer-generated random table approach. The groups were organized based on the type of mouthwash used. Allocation concealment was achieved through a sealed envelope method. Envelopes containing group assignments were prepared in advance and only opened by the researcher after participants had been enrolled, ensuring that allocation concealment was maintained and the assignment process remained unbiased.

The first group, Group A (the test group), with 90 participants, received a commercially available aloe vera mouthwash (KIN, 500 milliliters) containing 0% alcohol, aloe barbadense leaf juice, aqua, sorbitol, sodium fluoride (226 ppm F-), glycerin, xylitol, and mint flavor (ASIN: B00L7QP7WM). The second group, Group B (the positive control group), with 90 participants, was given a placebo distilled water mouthwash. The third group, Group C (the negative control group), with 90 participants, was given a 0.2% chlorhexidine mouthwash (500 milliliters), as described by Pattnaik et al. [3].

The mouthwashes were administered in identical containers with opaque coverings to ensure single blinding. Each participant was given written instructions on how to use the mouth rinse properly. All three groups received the exact oral hygiene instructions during the study, except for using the allocated mouth rinse. A container bearing an indistinguishable appearance was provided to every participant, which contained 500 ml of the mouthwash solution. A calibrated measuring cup featuring a 10 ml capacity was furnished to guarantee a precise dosage. All participants were directed to cleanse their mouths twice daily for one minute with 10 ml of the designated solution across all groups. The prescribed rinsing routine was to be executed regularly for 30-45 seconds, once in the morning and once before nightfall, for 30 days. Furthermore, participants were instructed to abstain from eating or cleansing with other substances for 30 minutes after using mouthwash.

Participants were prohibited from using additional oral hygiene products or procedures during the study, including interdental simulators or dental floss. On the 15^th^ and 30^th^ days following baseline, clinical evaluations of the GI and PI were performed and compared to the assessment of gingivitis and dental plaque at baseline. Participants were directed to return their bottles to document the leftover rinse solution, guaranteeing adherence to the rinsing process. All subjects followed the rinsing instructions precisely, with no variations in the frequency or amount of mouthwash.

Statistical analysis

The collected data underwent statistical analysis using IBM SPSS Statistics software for Windows, version 26, developed by IBM Corporation, Armonk, NY. Categorical variables were analyzed using chi-square and Fisher's exact tests. Repeated measures analysis was conducted on consecutive continuous variables, followed by multiple comparisons using a post hoc test. A p-value of 0.05 or below signifies statistical significance.

## Results

The study involved 270 healthy participants divided into three groups: 90 received aloe vera, 90 received chlorhexidine, and 90 received a placebo. There were no statistically significant differences in age (p = 0.06) or gender (p = 0.9) between the study groups (Table 1).

**Table 1:** Demographic characteristics of the study groups

Variable	Study groups						p-value
	Aloe vera		Chlorhexidine		Placebo		
	No.	%	No.	%	No.	%	
Age							0.06
<20 years	1	1.1	8	8.9	9	10.0	
20-29 years	31	34.4	40	44.4	35	38.9	
30-39 years	46	51.1	29	32.2	36	40.0	
≥40 years	12	13.3	13	14.4	10	11.1	
Gender							0.9
Male	43	47.8	42	46.7	44	48.9	
Female	47	52.2	48	53.3	46	51.1	

The study groups had no significant differences regarding the baseline PI (p = 0.7) and GI (p = 0.3) (Table 2).

**Table 2:** Baseline oral health measures of the study groups GI:  gingival index; PI: plaque index

Variable	Study groups						p-value
	Aloe vera		Chlorhexidine		Placebo		
	No.	%	No.	%	No.	%	
Baseline PI							0.7
Fair	47	52.2	51	56.7	46	51.1	
Bad	43	47.8	39	43.3	44	48.9	
Baseline GI							0.3
Mild	2	2.2	6	6.7	5	5.6	
Moderate	86	95.6	79	87.8	83	92.2	
Severe	2	2.2	5	5.6	2	2.2	

After a 30-day treatment with aloe vera mouthwash, there were significant decreases in the mean of GI and PI (p<0.001). Substantial reductions in the GI and PI were distinguished following 30 days of chlorohexidine mouthwash use (p<0.001). Similarly, using placebo mouthwash for 30 days resulted in prominent reductions in both GI and PI (p<0.001) (Table 3).

**Table 3:** Distribution of oral health measures according to study durations for different study groups

Study groups	Study durations			p-value
	Baseline	Day 15	Day 30	
	Mean ±SD	Mean ±SD	Mean ±SD	
Aloe vera group				
Plaque index	1.9±0.3	1.6±1.5	1.07±0.2	<0.001
Gingival index	1.5±0.2	1.1±0.2	0.7±0.2	<0.001
Chlorhexidine group				
Plaque index	1.9±0.4	1.5±0.3	1.03±0.2	<0.001
Gingival index	1.5±0.3	1.09±0.2	0.6±0.2	<0.001
Placebo group				
Plaque index	1.9±0.3	1.7±0.3	1.6±0.3	<0.001
Gingival index	1.5±0.3	1.4±0.3	1.3±0.3	<0.001

By post hoc analysis, the means of PI index for participants with aloe vera mouthwash and chlorohexidine mouthwash were not significantly different (p = 0.8). In contrast, the means of PI index for participants with aloe vera mouthwash were considerably lower than the PI means of participants with placebo mouthwash (p<0.001), and the means of PI index for participants with chlorohexidine mouthwash were significantly lower than the PI means of participants with placebo mouthwash (p<0.001) (Table 4).

**Table 4:** Multiple comparisons of the plaque index between the study groups

Multiple Comparisons						
Study groups	Compared groups	Mean difference	Std. error	p-value	95% Confidence interval	
					Lower bound	Upper bound
Aloe vera	Chlorhexidine	0.03	0.06	0.8	-0.11-	0.17
	Placebo	-0.23-	0.06	<0.000	-0.38-	-0.09-
Chlorhexidine	Aloe vera	-0.03-	0.06	0.8	-0.17-	0.11
	Placebo	-0.26-	0.06	<0.000	-0.41-	-0.12-
Placebo	Aloe vera	0.23	0.06	<0.000	0.09	0.38
	Chlorhexidine	0.26	0.06	<0.000	0.12	0.41

By post hoc analysis, the means of GI index for participants with aloe vera mouthwash and those with chlorohexidine mouthwash were not significantly different (p = 0.6). In contrast, the means of GI index for participants with aloe vera mouthwash was considerably lower than the GI means of participants with placebo mouthwash (p<0.001), and the means of GI index for participants with chlorohexidine mouthwash were markedly lower than the GI means of participants with placebo mouthwash (p<0.001) (Table 5). 

**Table 5:** Multiple comparisons of the gingival index between the study groups

Multiple Comparisons						
Study groups	Compared groups	Mean difference	Std. error	p-value	95% Confidence interval	
					Lower bound	Upper bound
Aloe vera	Chlorhexidine	0.03	0.041	0.6	-0.06-	0.13
	Placebo	-0.29-	0.041	<0.000	-0.38-	-0.19-
Chlorhexidine	Aloe vera	-0.03-	0.041	0.6	-0.13-	0.06
	Placebo	-0.32-	0.041	<0.000	-0.42-	-0.22-
Placebo	Aloe vera	0.29	0.041	<0.000	0.19	0.38
	Chlorhexidine	0.32	0.041	<0.000	0.22	0.42

There was a highly significant difference in PI on the 30^th^ day between study groups (p<0.001); 25.6% of aloe vera participants had healthy PI, 43.3% of chlorohexidine participants had healthy PI, and only 5.6% of placebo participants had healthy PI. There was a highly significant difference in GI on the 30th day between the study groups (p<0.001); 95.6% of aloe vera participants had mild gingivitis, and 96.7% of chlorohexidine participants had mild gingivitis, while only 27.8% of placebo participants had mild gingivitis (Table 6).

**Table 6:** Distribution of oral health measures on the 30th day according to the study groups PI: plaque index; GI: gingival index

Variable	Study groups						p-value
	Aloe vera		Chlorhexidine		Placebo		
	No.	%	No.	%	No.	%	
PI on the 30^th^ day							<0.001
Healthy	23	25.6	39	43.3	5	5.6	
Fair	67	74.4	50	55.6	76	84.4	
Bad	0	-	1	1.1	9	10.0	
GI on the 30^th^ day							<0.001
Mild	86	95.6	87	96.7	25	27.8	
Moderate	4	4.4	3	3.3	64	71.1	
Severe	0	-	0	-	1	1.1	

## Discussion

Over the past few years, there has been a noticeable shift in the dentistry field toward utilizing herbal and natural products for oral disease treatment. This is due to the antimicrobial properties of many plant-based products, which offer alternative options to traditional antibiotics and antimicrobial agents [15]. Furthermore, herbal remedies present a lower risk of long-term side effects when compared to pharmaceuticals [16]. Aloe vera, for instance, is acknowledged for its potent antibacterial properties, effectively fighting bacteria and helping to prevent plaque accumulation and periodontal diseases [1,16].

The study revealed a significant decrease in the mean scores of the gingival and plaque indices after a 30-day treatment with aloe vera mouthwash (p<0.001). The findings align with the quasi-experimental study by Hamonari et al. [2]; it showed reduced gingival and plaque indices associated with periodontal disease. In addition, a recent randomized controlled experiment by Kamath et al. [4] confirmed these results by comparing the antiplaque and anti-gingivitis efficacy of chlorhexidine and aloe vera mouthwash in patients with fixed orthodontics.

An additional double-blind placebo-controlled clinical investigation conducted in Iran, as reported by Yaghini et al., demonstrated a reduction in the mean values of the gingival and plaque indices following two weeks of Aloe vera mouthwash usage [17].

This study used chlorhexidine-containing mouthwash as a negative control, following the approach of several prior studies that compared it with other mouthwash products [3,4,18]. Chlorhexidine is highly regarded as the most effective treatment because of its outstanding ability to prevent plaque buildup and fight against microorganisms [3,4,18,19]. According to the current study, there was a statistically significant reduction in the means of the GI and PI following a 30-day treatment with chlorohexidine mouthwash (p<0.001). The results of this research align with the findings of a study conducted in the United States of America by Sreenivasan and Prasad; the study reported that within two weeks, the use of chlorhexidine mouthwash led to substantial decreases in both the GI and PI [19].

This study observed a significant reduction in the mean GI and PI after 30 days of using a placebo mouthwash with a p<0.001. This finding aligns with the results of the Gupta et al. double-blind, randomized controlled trial in India, which also reported significant decreases in GI and PI after 30 days of using a placebo mouthwash [10]. Furthermore, a randomized controlled trial in China by Xu et al. found that daily water mouthwash reduced GI and PI by altering oral microbiota from anaerobic to aerobic phenotypes [20].

Upon examination of the investigation findings, it was concluded that there was no statistically significant difference (p=0.8) in the average PI values between individuals who used chlorhexidine mouthwash and those who used aloe vera mouthwash. This outcome aligns with a study conducted in India by Yeturu et al. [21], which showed a significant decrease in PI after 15 days of using aloe vera and chlorhexidine mouthwashes, with no noteworthy differences.

However, the study noted that the mean PI index for individuals using aloe vera and chlorhexidine mouthwashes was significantly lower than those using a placebo mouthwash (p<0.001). This finding is consistent with a randomized controlled trial conducted in Syria by Alnouri et al. [18], which found a significant difference in PI between children using aloe vera and chlorhexidine mouthwashes compared to those using a placebo. Additionally, this aligns with the conclusions of a review study in Iran by Poorkazemi et al. [22], which suggested the potential of aloe vera mouthwash to reduce the GI to a level similar to that of chlorhexidine mouthwash.

The research indicated that participants who used mouthwash containing aloe vera and chlorhexidine had significantly lower GI scores than those who used a placebo mouthwash (p<0.001). This finding is consistent with Poorkazemi et al.'s narrative review [22], which reported the effectiveness of aloe vera in reducing periodontal indices compared to chlorhexidine. Additionally, the results are in line with the conclusions of Vangipuram et al. from their randomized controlled trial conducted in India, which demonstrated the superior effectiveness of a mouthwash containing chlorhexidine and aloe vera in reducing the GI compared to a placebo [1].

Furthermore, the study found a significant difference in the PI on the 30^th^ day among study groups (p<0.001): 25.6% of aloe vera participants had healthy PI, 43.3% of chlorohexidine participants had healthy PI, and only 5.6% of placebo participants had healthy PI. The findings are consistent with those of Al-Maweri et al., who conducted a comprehensive review to assess the efficacy of mouthwash containing aloe vera against gingival inflammation and plaque. The review found that aloe vera mouthwash is as beneficial as chlorhexidine in reducing gingival inflammation. However, it could be more efficacious [11].

The study found a significant difference in GI on the 30^th^ day between trial groups (p<0.001), with 95.6% of aloe vera participants and 96.7% of chlorohexidine participants experiencing mild gingivitis, compared to only 27.8% of the placebo group. These results are consistent with Parkar et al.'s randomized controlled trial study in India, which revealed that chlorohexidine mouthwash was more efficient than aloe vera mouthwash in reducing gingivitis [23].

Limitations of the study

Firstly, the short duration limits the ability to observe long-term effects and outcomes. Without an extended follow-up period, it is difficult to determine the sustained impact of the interventions or changes being studied. Secondly, conducting the study at only two centers may not account for variations in patient populations and clinical practices across different locations. Differences in demographic characteristics, healthcare infrastructure, and clinical protocols between centers could influence the results, and the study might not capture these variations.

Strength of the study

The study has several strengths. It uses a randomized controlled trial design, the gold standard in clinical research, ensuring reliable and robust results. Including a placebo group allows for a clear comparison between aloe vera and chlorhexidine mouthwashes, isolating their specific effects. Objective measures, such as plaque and gingival index scores, enhance the validity of the findings. The data collection methods were rigorously applied. Additionally, the large sample size of 270 participants improves the statistical power and generalizability of the results. The single-masked design further adds to the study's strength by minimizing bias.

## Conclusions

The study concluded that aloe vera mouthwash supports its anti-inflammatory and antimicrobial properties, potentially preventing plaque formation. The results indicate that aloe vera mouthwash effectively reduces plaque accumulation and gingival indices, leading to a decrease in gum inflammation and bleeding.

This study suggests considering aloe vera mouthwash as an alternative treatment for periodontal diseases, particularly for patients seeking natural or less potent alternatives to traditional chemical treatments. Further research with larger sample sizes and varying trial durations is recommended to confirm its effectiveness in preventing periodontal complications.
